# Madelung's disease with myopathy

**DOI:** 10.4103/0972-2327.53086

**Published:** 2009

**Authors:** C. J. Suresh Chandran, Y. R. Godge, P. J. Oak, S. H. Ravat

**Affiliations:** Department of Neurology, Seth GS Medical College and KEM Hospital, India

**Figure 1:**
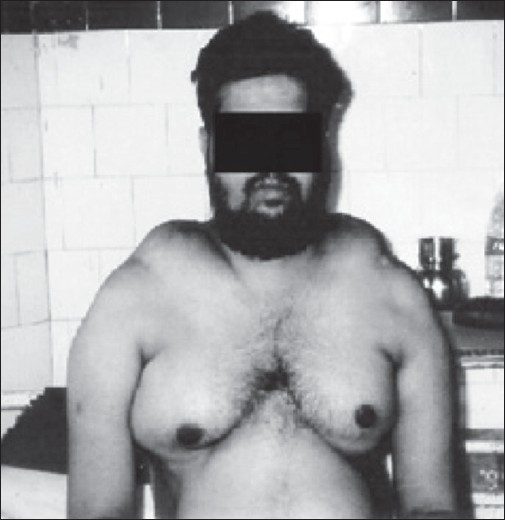
Shows symmetrical lipomatous deposits over both shoulders and breasts

**Figure 2:**
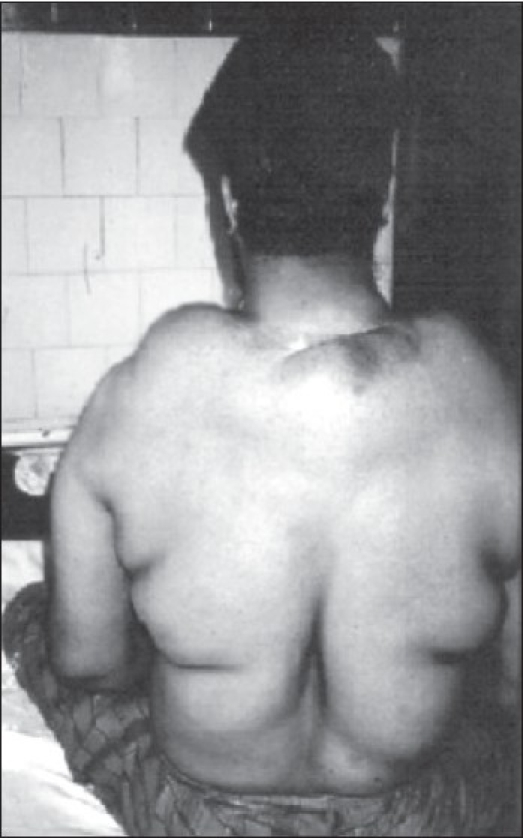
Shows midline lipomatous deposit [with scar of previous surgery] at the nape of neck and symmetrical deposits over shoulder, dorsal and lumbar regions

A 32-year-old male presented to us 2 years back with a 5-year history of multiple swellings over the neck, shoulder, chest, and trunk, associated with proximal weakness of the limb muscles. He had first noticed a small swelling over the lower part of the back of his neck 5 years ago; a biopsy had revealed lipoma. The weakness started symmetrically in the proximal muscles of the lower limbs and was followed, 6 months later, by proximal weakness of the upper limbs. Clinical examination revealed multiple symmetric lipomatous deposits over the shoulders, neck, chest, and trunk [Figures 1 and 2]. He had grade 3 power in the proximal muscles of the lower limbs and grade 4 power in the proximal muscles of the upper limb. The rest of the nervous system examination was normal.

Routine blood and urine investigations were normal. Serum CPK was 280 IU/L Fasting serum lipid profile, serum uric acid, and fasting and postprandial blood sugars were normal. Thyroid function test was normal. Serum lactate was 71.40 mg/dl (normal range: 5.70-22.00 mg/dl) and serum pyruvate was low at 0.20 mg/dl (normal range: 0.30-0.70 mg/dl). The lactate to pyruvate ratio was high (357:1). Nerve conduction study showed normal motor and sensory conductions. EMG did not show any spontaneous activities, but revealed small brief polyphasics in the proximal muscles of the upper and lower limbs, with early recruitment; this was suggestive of a primary muscle disease. CT chest and abdomen did not reveal any mediastinal or retroperitoneal lipomatosis. The patient refused a muscle biopsy. A diagnosis of Madelung's disease (type 1) was made. The patient was managed conservatively. His weakness progressed over time and at present, 7 years from the onset of symptoms, he is bedridden and fully dependent on others for the activities of daily living.

Madelung's disease [multiple symmetric lipomatosis (MSL), benign symmetric lipomatosis, or Launois-Bensaude syndrome] is characterized by subcutaneous accumulation of nonencapsulated adipose tissue in the neck, face, trunk, and limbs. Unlike the usual lipomas, these fatty masses do not have distinct boundaries and are not enclosed within a membranous capsule. Because of this characteristic and the absolute symmetry of this condition, it is often dismissed as simple obesity. It is commonly seen in middle-aged alcoholic males, though occasionally is reported in females and non-alcoholic males. Massive symmetric deposition of fat becomes cosmetically deforming in the parotid regions (hamster cheeks), cervical region (horse collar), posterior neck (buffalo hump), and submental region (Madelung's collar). Madelung's disease is associated with alcoholic liver disease, macrocytic anemia, proximal myopathy, somatic and autonomic neuropathy, and central nervous manifestations like ataxia, mitochondrial encephalopathy, etc.[1–4] Hyperlipoproteinemia, hyperuricemia, and impaired glucose tolerance test have also been described in this disease.[2] Rare complications include mediastinal and retroperitoneal lipomatosis.[3] Two types of lipomatosis based on the distribution of these fat deposits have been described. In type 1, circumscribed masses of fatty tumors protrude from an otherwise lean body. The lipomatous tissue in type 2 diffuses extensively into the subcutaneous fat layer, giving the patients an appearance of simple obesity.[5] Carlsen suggested the presence of a third type (type 3), a congenital form, in which the lipomatosis is mainly located around the trunk.[6] Male patients are characterized by the submental deposition of fat and the type 1 pattern. Females have obesity-like appearance typical of type 2 and lack submental deposition. The typical morphological feature in women is the fat deposition in the proximal arms and legs, with sparing of the distal limbs, giving a characteristic ‘football player’ appearance.[7]

The pathogenesis of this disease is unknown. Suggested mechanisms include a defect in the lipolytic pathway of the fat cell or an abnormal lipogenesis induced by catecholamines.[2] The lipomas are thought to be the result of defective brown adipose tissue. The type 2 MSL might have a place on the edge of the obesity spectrum. Adipogenesis in MSL is not a consequence of energy excess but is an active hyperplastic proliferation of subcutaneous adipose tissue.[8] Recent studies have shown defects of the mitochondrial respiratory chain and deletions and point mutations of mitochondrial DNA in patients suffering from this disease.[4,9] Abstinence from alcohol may prevent further progression in the size of the fat deposits. Dietary management does not help. Recent reports suggest a possible role for pharmacological treatment (with fibrates, magnesium, and pyridoxine) for arresting the progression of the disease.[10,11] Surgical options include lipectomy or liposuction, especially when the lesions are large and cause pressure effects on adjacent structures. Long-term complications include mediastinal compression syndromes, obstructive sleep apnea, myopathy, polyneuropathy, and malignant transformation of fatty deposits.

Our patient of Madelung's disease was a young non-alcoholic male and had significant proximal myopathy. To the best of our knowledge, this is the first reported case of Madelung disease with proximal myopathy from our country. Possible causes of myopathy in Madelung's disease include alcoholic myopathy and mitochondrial myopathy. Being non-alcoholic, our patient probably has a mitochondrial myopathy (the high lactate to pyruvate ratio is supportive of this possibility). Madelung's disease is ‘sight diagnosis.’ But all patients should be subjected to a detailed evaluation to detect any associated metabolic and neurological complications.
